# Utility of a Lateral Flow Immunoassay (LFI) to Detect *Burkholderia pseudomallei* in Soil Samples

**DOI:** 10.1371/journal.pntd.0005204

**Published:** 2016-12-14

**Authors:** Patpong Rongkard, Viriya Hantrakun, Sabine Dittrich, Prapaporn Srilohasin, Premjit Amornchai, Sayan Langla, Cherry Lim, Nicholas P. J. Day, David AuCoin, Vanaporn Wuthiekanun, Direk Limmathurotsakul

**Affiliations:** 1 Mahidol-Oxford Tropical Medicine Research Unit, Faculty of Tropical Medicine, Mahidol University, Bangkok, Thailand; 2 Lao-Oxford-Mahosot Hospital-Wellcome Trust Research Unit, Mahosot Hospital, Vientiane, Lao PDR; 3 Centre for Tropical Medicine and Global Health, Nuffield Department of Medicine, University of Oxford, Headington, Oxford, United Kingdom; 4 Foundation for Innovative New Diagnostics (FIND), Geneva, Switzerland; 5 Department of Microbiology and Immunology, University of Nevada School of Medicine, Reno, Nevada, United States of America; 6 Department of Tropical Hygiene, Faculty of Tropical Medicine, Mahidol University, Bangkok, Thailand; Institut Pasteur, FRANCE

## Abstract

**Background:**

Culture is the gold standard for the detection of environmental *B*. *pseudomallei*. In general, soil specimens are cultured in enrichment broth for 2 days, and then the culture broth is streaked on an agar plate and incubated further for 7 days. However, identifying *B*. *pseudomallei* on the agar plates among other soil microbes requires expertise and experience. Here, we evaluate a lateral flow immunoassay (LFI) developed to detect *B*. *pseudomallei* capsular polysaccharide (CPS) in clinical samples as a tool to detect *B*. *pseudomallei* in environmental samples.

**Methodology/Principal Findings:**

First, we determined the limit of detection (LOD) of LFI for enrichment broth of the soil specimens. Soil specimens (10 grams/specimen) culture negative for *B*. *pseudomallei* were spiked with *B*. *pseudomallei* ranging from 10 to 10^5^ CFU, and incubated in 10 ml of enrichment broth in air at 40°C. Then, on day 2, 4 and 7 of incubation, 50 μL of the upper layer of the broth were tested on the LFI, and colony counts to determine quantity of *B*. *pseudomallei* in the broth were performed. We found that all five soil specimens inoculated at 10 CFU were negative by LFI on day 2, but four of those five specimens were LFI positive on day 7. The LOD of the LFI was estimated to be roughly 3.8x10^6^ CFU/ml, and culture broth on day 7 was selected as the optimal sample for LFI testing. Second, we evaluated the utility of the LFI by testing 105 soil samples from Northeast Thailand. All samples were also tested by standard culture and quantitative PCR (qPCR) targeting *orf2*. Of 105 soil samples, 35 (33%) were LFI positive, 25 (24%) were culture positive for *B*. *pseudomallei*, and 79 (75%) were qPCR positive. Of 11 LFI positive but standard culture negative specimens, six were confirmed by having the enrichment broth on day 7 culture positive for *B*. *pseudomallei*, and an additional three by qPCR. The LFI had 97% (30/31) sensitivity to detect soil specimens culture positive for *B*. *pseudomallei*.

**Conclusions/Significance:**

The LFI can be used to detect *B*. *pseudomallei* in soil samples, and to select which samples should be sent to reference laboratories or proceed further for bacterial isolation and confirmation. This could considerably decrease laboratory workload and assist the development of a risk map for melioidosis in resource-limited settings.

## Author Summary

*Burkholderia pseudomallei* is an environmental Gram-negative bacillus and the causative agent of melioidosis. Culture and PCR assays are standard diagnostic tools used to detect *B*. *pseudomallei* in the environment. However, those tests require experienced microbiologists and are regularly conducted only in a few research laboratories worldwide. In this study, we demonstrated that the prototype lateral flow immunoassay (LFI) developed to detect *B*. *pseudomallei* capsular polysaccharide (CPS) in clinical samples could be used to detect *B*. *pseudomallei* in environmental samples. We found that the LFI can be used to detect *B*. *pseudomallei* in experimentally spiked soil specimens. Next, we evaluated the sensitivity of LFI using 105 soil samples collected in Northeast Thailand. We found that the LFI had high sensitivity to detect *B*. *pseudomallei* in the soil. We propose that the LFI could be used to detect environmental *B*. *pseudomallei* in resource-limited settings. Soil samples positive for LFI could be sent to reference laboratories for confirmation with culture or molecular methods. The use of LFI could assist in the development of a global risk map for melioidosis.

## Introduction

The Gram-negative *Burkholderia pseudomallei* is a soil-dwelling organism and also the cause of melioidosis, an often fatal infectious disease [1,2]. Melioidosis can be difficult to diagnose due to its diverse clinical manifestations. The diagnostic confirmation relies on microbiological culture, which is often unavailable in resource-restricted regions of the world [3]. Even with such facilities, *B*. *pseudomallei* may be dismissed as a culture contaminant [4], or be misidentified by standard identification methods including API 20NE and automated bacterial identification systems [5,6]. The disease occurs as a result of skin inoculation, inhalation and ingestion of environmental *B*. *pseudomallei* [7]. The organism is intrinsically resistant to a wide range of antimicrobials, and treatment with ineffective antimicrobials may result in case fatality rates (CFRs) exceeding 70% [8,9]. A recent spatial modeling study estimated there to be 165,000 human melioidosis cases per year worldwide, of which 89,000 die [10]. The study also estimated that melioidosis is severely underreported in the 45 countries in which it is known to be endemic and that *B*. *pseudomallei* is likely present in a further 34 countries in which melioidosis has never been reported [10].

Defining the distribution of *B*. *pseudomallei* in countries where *B*. *pseudomallei* is likely present but melioidosis has never been reported is important, since this will provide policy makers with evidence for raising awareness of this disease among healthcare workers and microbiology laboratories in these areas [11]. Environmental sampling can be used to identify areas where people are at risk even before cases are recognized. For example, the first environmental survey around Vientiane City (Lao PDR) in 1998 demonstrated the presence of *B*. *pseudomallei* prior to the recognition of human disease [12]. This environmental finding resulted in an effort to identify *B*. *pseudomallei* from clinical specimens, with the first case of melioidosis being identified in 1999 [13]. Since then, more than 920 culture-positive melioidosis patients have been identified in Lao PDR [14]. Environmental sampling can also be used to confirm the endemicity of melioidosis after identifying melioidosis cases in new areas. Recent findings include the detection of environmental *B*. *pseudomallei* after case reports in Gabon [15] and Bangladesh [16].

Culture and PCR assays are commonly used to detect *B*. *pseudomallei* in the environment, but both tests require experienced microbiologists and are only available in a few research laboratories worldwide. For the culture method, soil specimens are initially cultured in selective broth for 2 days, and then the upper layer of the broth is streaked on an agar plate and incubated for a further 7 days [11]. Identifying *B*. *pseudomallei* on agar plates among other soil microbes is time-consuming and requires expertise and experience. For effective molecular detection, soil specimens are also first enriched in selective broth for 2 days prior to nucleic acid amplification [17,18]. Despite the higher sensitivity than culture, PCR assays also require experienced personnel and positive controls, and are relatively costly for resource-limited settings.

Recently, a lateral flow immunoassay (LFI) was developed to detect *B*. *pseudomallei* capsular polysaccharide (CPS) in clinical samples [19]. In a pilot study, the LFI was shown to have 100% sensitivity for all urine samples containing *B*. *pseudomallei* greater than 1.2×10^4^ CFU/ml [19]. Here, we evaluate the LFI as a tool to detect *B*. *pseudomallei* in the enrichment broth of the environmental samples. We found that the LFI had high sensitivity in our setting.

## Methods

### Determining the limit of detection (LOD) of LFI to detect *B*. *pseudomallei* in enrichment media on experimental soil samples

*B*. *pseudomallei* wild-type strain E08 and 300 grams of soil culture negative for *B*. *pseudomallei* collected from Nakorn Ratchasima, Northeast Thailand, were used. The strain E08 was selected as the strain has been frequently used as a representative strain of *B*. *pseudomallei* from the environment [20–22]. Soil specimens (10 gram/specimen) were spiked with *B*. *pseudomallei* at an inoculum of 10 to 10^5^ colony forming units (CFU) per 10 grams of soil, and incubated in 10 ml of enrichment broth (threonine-basal salt solution plus colistin at 50 mg/liter [TBSS-C50]) in air at 40°C. Unspiked soil samples and soil samples sterilised by autoclaving were included as negative controls. Soil samples sterilised by autoclaving, TBSS-C50 and sterile distilled water, all spiked with *B*. *pseudomallei*, were also included as positive controls. After incubation for 2, 4 and 7 days, the upper layer of enrichment broth were tested by LFI, and evaluated by culture for quantity of *B*. *pseudomallei*. A total of nine soil specimens were used to cover the range of inoculum from 10 to 10^5^ CFU per 10 grams of soil, while five specimens was used for the lowest inoculum of 10 CFU per 10 grams of soil (Table 1).

**Table 1:** LFI results and quantitative *B*. *pseudomallei* count of enrichment broth of the experimental soil specimens.

Tube No.	Specimens	*B*. *pseudomallei* Inoculation	Enrichment broth on day	LFI test	Quantitative *B*. *pseudomallei* count (CFU/ml)	Notes
1	soil 10 g ^a^	10^5^ CFU	0	ND	3.8 x 10^3^	Expected to have 10^4^ CFU/ml
			**2**	**Positive**	4.9 x 10^7^	
			4	Positive	1.3 x 10^8^	Fungi 1+
			7	Positive	4.5 x 10^7^	Fungi 2+
2	soil 10 g ^a^	10^4^ CFU	0	ND	5.7 x 10^2^	Expected to have 10^3^ CFU/ml
			**2**	**Positive**	2.6 x 10^7^	
			4	Positive	8.6 x 10^6^	Fungi 1+
			7	Positive	1.2 x 10^5^	Fungus 3+
3	soil 10 g ^a^	10^3^ CFU	0	ND	35	Expected to have 10^2^ CFU/ml
			**2**	**Positive**	9.1 x 10^6^	
			4	Positive	2.3 x 10^7^	Fungi 1+
			7	Positive	6.1 x 10^4^	Fungus 4+
4	soil 10 g ^a^	10^2^ CFU	0	ND	10	Expected to have 10 CFU/ml
			**2**	**Positive**	3.8 x 10^6^	
			4	Positive	5.5 x 10^6^	Fungi 1+
			7	Positive	6.4 x 10^6^	Fungi 2+
5	soil 10 g ^a^	10 CFU	0	ND	0	Expected to have 1 CFU/ml
			2	Negative	8.4 x 10^5^	
			**4**	**Positive**	7.6 x 10^5^	Fungi 1+
			7	Positive	9.0 x 10^3^	Fungi 2+
6	soil 10 g ^a^	10 CFU	0	ND	0	Expected to have 1 CFU/ml
			2	Negative	1.0 x 10^4^	
			4	Negative	2.1 x 10^6^	
			**7**	**Positive**	3.0 x 10^7^	Fungi 1+
7	soil 10 g ^a^	10 CFU	0	ND	0	Expected to have 1 CFU/ml
			2	Negative	2.0 x 10^4^	
			4	Negative	1.9 x 10^6^	
			**7**	**Positive**	1.5 x 10^7^	Fungi 1+
8	soil 10 g ^a^	10 CFU	0	ND	0	Expected to have 1 CFU/ml
			2	Negative	9.0 x 10^3^	
			4	Negative	3.0 x 10^5^	
			**7**	**Positive**	1.8 x 10^6^	Fungi 1+
9	soil 10 g ^a^	10 CFU	0	ND	0	Expected to have 1 CFU/ml
			2	Negative	3.0 x 10^3^	
			4	Negative	1.0 x 10^5^	Fungi 1+
			7	Negative	2.0 x 10^4^	Fungi 1+

ND = Not done. Notes show expected quantitative *B*. *pseudomallei* count of enrichment broth on day 0, and fungi present on agar plates (1+, 2+, 3+ and 4+ represent the amount of fungi).

^a^ Soil culture negative for *B*. *pseudomallei* was used. Soil were enriched with 10 ml of TBSS-C50 (threonine-basal salt solution plus colistin 50 mg/L) and incubated in air at 40°C for 7 days.

The Active Melioidosis Detect (AMD) LFI developed by InBios (Seattle, USA) was used [19]. The evaluated LFI uses a monoclonal antibody (mAb) 4C4 targeting the CPS of *B*. *pseudomallei*. MAb 4C4 was sprayed onto a nitrocellulose membrane strip for the test line, and goat anti-chicken IgY (Lampire Biological laboratories, Pennsylvania, USA) was sprayed on the same membrane for the control line. The conjugate pad contained dried 40 nm gold particles conjugated with mAb 4C4 as well as a small amount of gold conjugated with chicken IgY (to react with the control line). The conjugate pad was treated with a borate-based buffer containing a small concentration of detergent and dried for later gold conjugate application. The sample application pad was also treated similarly and dried. The LFI was assembled by combining the sprayed membrane, conjugate pad, and sample pad on top of an adhesive plastic backing. The LFI was stored in air at room temperature until use. A total of 50 μl of the upper layer of the broth was mixed with two drops of lysis buffer and two drops of chase buffer in a clean tube, in which the LFI pad was dipped. After 15 minutes at room temperature, the LFI results were read.

The quantitative count of *B*. *pseudomallei* in the enrichment broth was performed as described previously with minor modifications [23]. In short, two 100 μl samples and two 10 μl samples of the upper layer of enrichment broth were spread onto four modified Ashdown’s agar plates using a rotary plater so that the individual colonies could be identified. All plates were then incubated in air at 40°C. The plates were inspected daily for 4 days, and *B*. *pseudomallei* was identified as previously described [11]. The LOD of LFI was estimated by comparing the quantitative *B*. *pseudomallei* results between samples with positive and negative LFI results. The quantitative counts of *B*. *pseudomallei* in specimens with high levels of fungus overgrowth were excluded. The optimal duration of enrichment for LFI testing was determined by selecting the duration that provided the highest proportion of LFI positivity.

### Evaluating utility of LFI to detect *B*. *pseudomallei* in the enrichment media of soil samples from Northeast Thailand

A total of 105 environmental soil samples were collected from 21 rice paddy fields located in 7 provinces (Burirum, Chaiyaphum, Khon Kaen, Loei, Nakhon Ratchasima, Nong Bua Lam Phu and Udon Thani) in Northeast Thailand during March and June 2015. Three villages per each province were randomly selected for study. We calculated that 105 samples were needed to estimate sensitivity of LFI with the 95% confidence interval of 15%. The randomization was performed using Stata version 14.0 (StataCorp LP, College Station, Texas). A rice field in each village was selected, and the first 5 soil samples collected in each rice field were used for the study. In each rice field, the soil samples was collected 2.5 meters apart. The soil sampling and culture was performed according to the consensus guidelines for environmental sampling of *B*. *pseudomallei* developed by DEBWorP [11] with slight modifications. In short, 10 grams of soil were collected at a depth of 30 cm. Each 10 grams of soils were incubated in 10 ml of TBSS-C50 enrichment broth (threonine-basal salt solution with 50 mg/L colistin) at 40°C in air for 7 days. After incubation for 2 and 7 days, 10 μl of the upper layer of enrichment broth were directly plated on Ashdown agar and incubated at 40°C in air further for 7 days [24], and *B*. *pseudomallei* was then identified as previously described [11]. In addition, 50 μl of the upper layer of enrichment broth on day 7 was tested on the LFI as described above and 1 ml of the upper layer of enrichment broth on day 7 was stored for qPCR assay. Culture of the upper layer of enrichment broth on day 7 is not normally conducted, but was performed in this study in order to evaluate the presence of *B*. *pseudomallei* in soil specimens which were negative by standard culture but LFI positive. Three microbiologists performed culture, LFI and qPCR independently, and they were blinded to the results of the other two tests.

### Detecting *B*. *pseudomallei* in the enrichment media by quantitative real-time PCR (qPCR)

A 1 mL aliquot of broth on day 7 was stored at -20°C until further processing. Prior to nucleic acid extraction, the broth was spun at 12,000g for 10 min, and all but the pellet and 200 μL of broth were removed. The remaining DNA pellet and broth were processed using the Qiagen Mini Kit using the bacterial extraction protocol, according to the manufacturers instructions. The qPCR assay targeting *orf2* of *B*. *pseudomallei* type III secretion system (TTS1) was performed as previously described [18].

### Statistical analysis

Utility of the LFI was determined by comparing the results between culture of broth on day 2, LFI of broth on day 7 and qPCR of broth on day 7. Culture of broth on day 2 was selected for the main comparison because it is the standard method used for environmental detection of *B*. *pseudomallei* in soil samples [11]. The degree of agreement between culture and LFI was expressed using the Kappa index and its *P* value. This describes the level of association, both positive and negative, beyond that caused by chance, as follows: 0.00–0.20, slight; 0.21–0.40, fair; 0.41–0.60, moderate; 0.61–0.80, substantial; 0.81–1.00, high.

## Results

### Limit of detection (LOD) of LFI to detect *B*. *pseudomallei* in the enrichment media

A total of nine soil specimens (10 gram/specimen) were spiked with *B*. *pseudomallei* at an inoculum of 10 to 10^5^ CFU per 10 grams of soil, and incubated in 10 ml of enrichment broth in air at 40°C (Table 1). Immediately after inoculation and mixing, we found that the quantitative count of *B*. *pseudomallei* in the broth ranged from 0% to 100% of the inoculum. All 0% results were from five specimens inoculated with 10 CFU of *B*. *pseudomallei* per 10 gram of soil (Table 1). The 100% result was the specimen inoculated with 100 CFU of *B*. *pseudomallei* per 10 gram of soil, with the quantitative count after inoculation similar to the expected concentration (10 CFU/ml; Table 1).

On day 2, the quantitative count of *B*. *pseudomallei* in the broth of five soil specimens spiked with 10 CFU ranged from 3x10^3^ to 8.4x10^5^ CFU/ml. Nonetheless, all of those five specimens were LFI negative (Table 1, Tube No. 5 to 9). On day 4, the quantitative count of *B*. *pseudomallei* in the broth of those five specimens ranged from 1x10^5^ to 2.1x10^6^ CFU/ml. Only one specimen (with a quantitative count 7.6x10^5^ CFU/ml) was LFI positive, while the remaining four were LFI negative. On day 7, *B*. *pseudomallei* count in the broth of those five specimens ranged from 9x10^3^ to 1.3x10^8^ CFU/ml. There was some difficulty obtaining quantitative counts in the two specimens with low counts (9x10^3^ and 2x10^4^ CFU/ml) due to overgrowth with fungi. Nonetheless, four specimens were LFI positive, while the remaining one with the count of 2x10^4^ CFU/ml was LFI negative (Table 1). S1 Table shows the results of all control specimens.

Using the quantitative counts of all the specimens, we found that the highest count that had a negative LFI result was 2.1x10^6^ CFU/ml (Tube 6), and the next higher count that had LFI positive was 3.8x10^6^ CFU/ml (Tube 4, Table 1). Therefore, we estimated that the LOD of the investigated LFI to detect *B*. *pseudomallei* in the enrichment broth of soil samples was about 3.8x10^6^ CFU/ml. As 80% of enrichment broth on day 7 of soil specimens spiked with 10 CFU of *B*. *pseudomallei* per 10 grams of soil were LFI positive while only 20% of those enrichment broth on day 4 were LFI positive, the enrichment broth on day 7 was selected as the optimal sample for LFI.

### Utility of LFI using soil samples from Northeast Thailand

**Fig 1:**
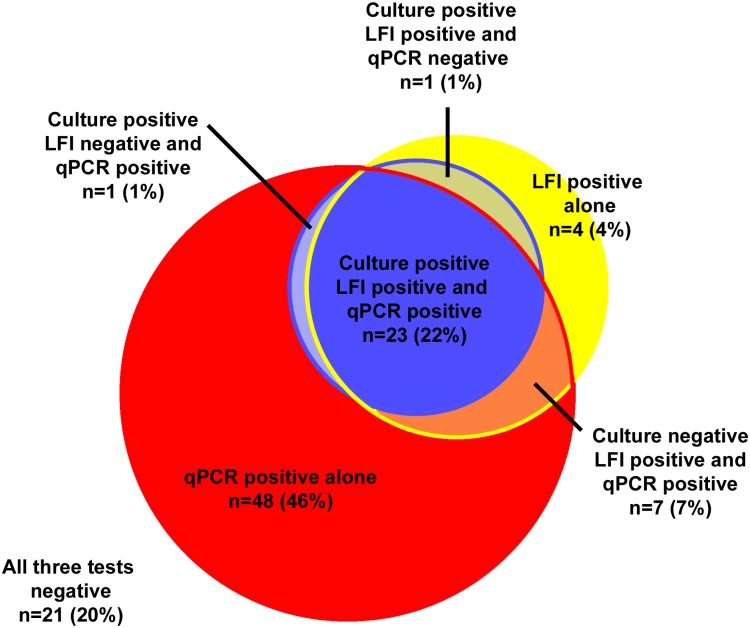
Venn diagram showing culture (blue), lateral flow immunoassay (LFI; yellow) and qPCR assay (red) results of 105 soil samples. Culture was performed according to the consensus guidelines for environmental sampling of *B*. *pseudomallei* developed by DEBWorP [11], in which soil specimens were enriched in the enrichment broth for 2 days and the upper layer of enrichment broth on day 2 was streaked on agar plates and observed daily. For the LFI and qPCR assay, soil specimens were enriched in the broth for 7 days and the broth on day 7 was used for the tests.

Of 105 soil samples collected in Northeast Thailand, 25 (24%) were culture positive for *B*. *pseudomallei* using the standard culture method, 35 (33%) were LFI positive, and 79 (75%) were qPCR positive (Fig 1). Agreement of results between the standard culture and LFI was substantial (Kappa index 0.72, p<0.001). Of 11 specimens LFI positive but the culture of broth on day 2 negative, six had broth on day 7 culture positive for *B*. *pseudomallei*, three were qPCR positive but broth on day 7 culture negative, and the other two were both qPCR and culture of broth on day 7 negative. We estimated that the LFI had 97% (30/31; 95% confidence interval 83%-99%) sensitivity for detection of soil specimens culture positive for *B*. *pseudomallei*.

## Discussion

This study demonstrates the utility of LFI as a tool to detect *B*. *pseudomallei* in soil samples. The LFI has a high sensitivity (97%) to detect soil specimens culture positive for *B*. *pseudomallei*. The test is very simple to use and does not require expertise or new equipment; therefore, LFI could be useful for inexperienced microbiologists who want to conduct an environmental survey in resource-limited settings. One explanation of the high sensitivity of the LFI is that we chose the enriched broth on day 7 as the specimen for LFI. Enrichment of soil specimens increases the concentration of *B*. *pseudomallei* in the broth exponentially, and the enrichment step (for 1–2 days) is already necessary for both culture and molecular detection [17,18]. We show that, for the evaluated LFI, enrichment of only 2 days is not sufficient, and 7 days are needed to increase the quantity of *B*. *pseudomallei* in the broth from as low as 1 CFU/ml (equivalent to 1 CFU/gram of soil) up to 3.8 x 10^6^ CFU/ml high enough to be detected.

Although culture is the gold standard for the detection of environmental *B*. *pseudomallei*, it requires expertise and experience. Colonies of *B*. *pseudomallei* on Ashdown agar plates can have seven morphologies [25] and be dismissed as other soil bacteria [4]. In addition, although Ashdown agar contains crystal violet and gentamicin to suppress the growth of other bacteria, overgrowth of other soil bacteria and fungi on agar plates is common [26], particularly after 7 days of incubation. In this study, we report the advantage of LFI for the detection of *B*. *pseudomallei* in soil samples as the reading is simple and can be performed by microbiologists otherwise not experienced with *B*. *pseudomallei*. Preliminary detection of *B*. *pseudomallei* in enrichment media of soil samples by LFI will also reduce the workload of at melioidosis reference laboratories by focusing on the confirmation of identified LFI positives rather than screening large sample numbers. This could enhance the development of a global risk map for melioidosis, particularly in areas without melioidosis reference laboratories [11].

The LOD of LFI in soil enrichment (3.8x10^6^ CFU/ml) is comparatively higher than its previously reported LOD in direct urine samples (1.2 x 10^4^ CFU/ml) [19]. This is probably due to the presence of other organisms and other substances in the soil enrichment.

The long total time required to complete the LFI test (7 days) is not a major problem as the total time required for the culture method is also 9 days. The culture method requires the soil specimen to be in the enrichment broth for 2 days, and then the upper layer of the broth is streaked on plates and incubated for a further 7 days. *B*. *pseudomallei* can survive well in soil kept at ambient temperature (24 to 32°C) and away from direct sunlight [11]. Therefore, culture and qPCR assays can be performed later from the soil specimens which are LFI positive. A study reported that the numbers of *B*. *pseudomallei* in soil increased moderately over 2 weeks when kept at 20°C [27]. The isolation of environmental *B*. *pseudomallei* provides definitive evidence that people in the areas sampled are at risk of melioidosis; therefore, it is important to use the test or a combination of tests with high accuracy rather than a rapid test with low accuracy.

The high proportion of specimens with were culture negative but qPCR positive (Fig 1) could be due to many reasons. First, culture results were based on enrichment of soil samples on day 2, while qPCR was based on that on day 7, resulting in higher number of *B*. *pseudomallei*. Second, qPCR has a lower detection limit compared to the culture method [18], and could also detect non-culturable *B*. *pseudomallei* [27]. In this study, qPCR confirmed the presence of *B*. *pseudomallei* in an additional three specimens that were culture negative but LFI positive. As qPCR is more sensitive than culture and LFI, the molecular methods should be considered if absence of the organism in the environment needs to be confirmed and resources are available [28]. Nonetheless, PCR assays for environmental samples require experienced molecular microbiologists and separate facilities, which are largely not available in areas where melioidosis could be highly endemic.

The LFI may have some limitations. First, the LFI could yield false positive results if a variant of *B*. *thailandensis* which produces CPS similar to *B*. *pseudomallei* is present in the soil. Second such *B*. *thalandensis* variants have been reported in soil from the USA (Texas) [29], Cambodia [30] and Laos [18], but not so far in soil from other areas. Second, the quantitative counts (CFU/ml) in our study could be underestimated due to overgrowth of fungi and other bacteria. Third, the use of LFI may lead to a long total time, as repeated bacterial culture would be needed for the confirmation of *B*. *pseudomallei* in the environment (15 days; 7 days of LFI plus 9 days of culture). If transportation of LFI-positive soil samples to reference laboratories is required, it would be preferred within 7-days of completing the LFI tests [27]. Sample site selection and total number of samples per site could be performed according to the consensus guidelines for environmental sampling of *B*. *pseudomallei* developed by DEBWorP [11]. Nonetheless, if rapid results are required, molecular methods should be considered [28]. Fourth, the utility of LFI may be different in other regions where the soil physicochemical properties are different, *B*. *pseudomallei* numbers lower and/or other organisms are present. Fifth, Our study sample size is not large. Further studies should also investigate the sensitivity and utility of the LFI to detect *B*. *pseudomallei* in soil outside Northeast Thailand.

In conclusion, we recommend that the LFI could be used to detect environmental *B*. *pseudomallei* in new areas, particularly when the investigation is conducted by microbiologists who have limited experience with the isolation and identification of *B*. *pseudomallei* from soil specimens. LFI positive soil specimens could then be sent to reference laboratories for bacterial isolation and confirmation. This could considerably decrease laboratory workload and assist in the development of a global risk map for melioidosis.

## Supporting Information

S1 Table LFI results and quantitative *B*. *pseudomallei* counts of enrichment broth of negative and positive controls. (DOCX)
