# Supplementary material for: Utility of a Lateral Flow Immunoassay (LFI) to Detect Burkholderia pseudomallei in Soil Samples
Source: PLoS Negl Trop Dis. 2016 Dec 14;10(12):e0005204. doi: 10.1371/journal.pntd.0005204 (PMC5156366; doi:10.1371/journal.pntd.0005204)
Supplement: S1 Table — (DOCX) [file pntd.0005204.s001.docx]

**S1 Table.** LFI results and quantitative *B. pseudomallei* counts of enrichment broth of negative and positive controls

| **Tube No.** | **Specimen** | ***B. pseudomallei***  **Inoculation** | **Enrichment broth on day** | **LFI test** | **Quantitative**  ***B. pseudomallei* count (CFU/ml)** |
| --- | --- | --- | --- | --- | --- |
| 10 | Sterile soil ^a^ | 0 CFU | 0 | ND | 0 |
|  |  |  | 2 | Negative | 0 |
|  |  |  | 4 | Negative | 0 |
|  |  |  | 7 | Negative | 0 |
| 11 | Sterile soil ^a^ | 10 CFU | 0 | ND | 0 |
|  |  |  | 2 | Positive | 1.5 x 10^7^ |
|  |  |  | 4 | Positive | 5.3 x 10^7^ |
|  |  |  | 7 | Positive | 1.3 x 10^8^ |
| 12 | TBSS-C50 ^b^ | 10 CFU | 0 | ND | 0 |
|  |  |  | 2 | Negative | 2.3 x 10^6^ |
|  |  |  | 4 | Positive | 1.7 x 10^8^ |
|  |  |  | 7 | Positive | 2.7 x 10^8^ |
| 14 | Distilled water ^b^ | 10 CFU | 0 | ND | 0 |
|  |  |  | 2 | Negative | 6.3 x 10^5^ |
|  |  |  | 4 | Negative | 6.7 x 10^5^ |
|  |  |  | 7 | Negative | 1.0 x 10^6^ |

ND = Not done

^a^ Soil was sterilized by autoclaving. Tube 10 and 11 were a negative and positive control, respectively. Soil was enriched with 10 ml of TBSS-C50 (threonine-basal salt solution plus colistin 50 mg/L) and incubated in air at 40°C for 7 days.

^b^ Tube 12-13 were used as positive controls without soil specimens.
